# Supplementary material for: System biology and network-based approach to identify the therapeutic signatures and potential inhibitors against polycystic lipomembranous osteodysplasia with Sclerosing Leukoencephalopathy
Source: PLoS One. 2026 Feb 20;21(2):e0343274. doi: 10.1371/journal.pone.0343274 (PMC12923142; doi:10.1371/journal.pone.0343274)
Supplement: S1 Fig — (DOCX) [file pone.0343274.s001.docx]

Supplementary information

System Biology and Network-Based Approach to Identify the Therapeutic Signatures and Potential Inhibitors Against Polycystic Lipomembranous Osteodysplasia with Sclerosing Leukoencephalopathy

|  |
| --- |

Bayan T. Bokhari ^1^*, Alaa M. Saleh ^1^, Hashim M. Aljohani ^2,3^, Wejdan Hussain Owaydhah ^4^, Mohammad Ahmad Alobaidy ^5^, Naief Dahran ^6^, Hind M Naffadi ^7^ and Alaa Abdulaziz Eisa ^2^*

^1^Department of Clinical Laboratory Sciences, Faculty of Applied Medical Sciences, Umm Al-Qura University, Makkah, Saudi Arabia. btbokhari@uqu.edu.sa, amsaleh@uqu.edu.sa

^2^Department of Clinical Laboratory Sciences, College of Applied Medical Sciences, Taibah University, Medina, Saudi Arabia. hsnani@taibahu.edu.sa, aeisa@taibahu.edu.sa

^3^Department of Pathology and Laboratory Medicine, College of Medicine, University of Cincinnati, Cincinnati, OH 45221, USA.

^4^Department of Basic Medical Sciences, Faculty of Medicine, Taibah University, Madinah, Saudi Arabia. wawedh@taibahu.edu.sa.

^5^Department of Anatomy, Faculty of Medicine, Umm Al-Qura University, Makkah P.O. Box 7607, Saudi Arabia. Maobaidy@uqu.edu.sa.

^6^Department of Basic Medical Sciences, College of Medicine, University of Jeddah, Jeddah, Saudi Arabia. ndahran@uj.edu.sa

^7^Department of Medical Genetics, faculty of medicine, Umm Al-Qura University, Makkah, Saudi Arabia. Hmnaffadi@uqu.edu.sa

***** Correspondence: Bayan T. Bokhari (btbokhari@uqu.edu.sa) and Alaa Abdulaziz Eisa (aeisa@taibahu.edu.sa)


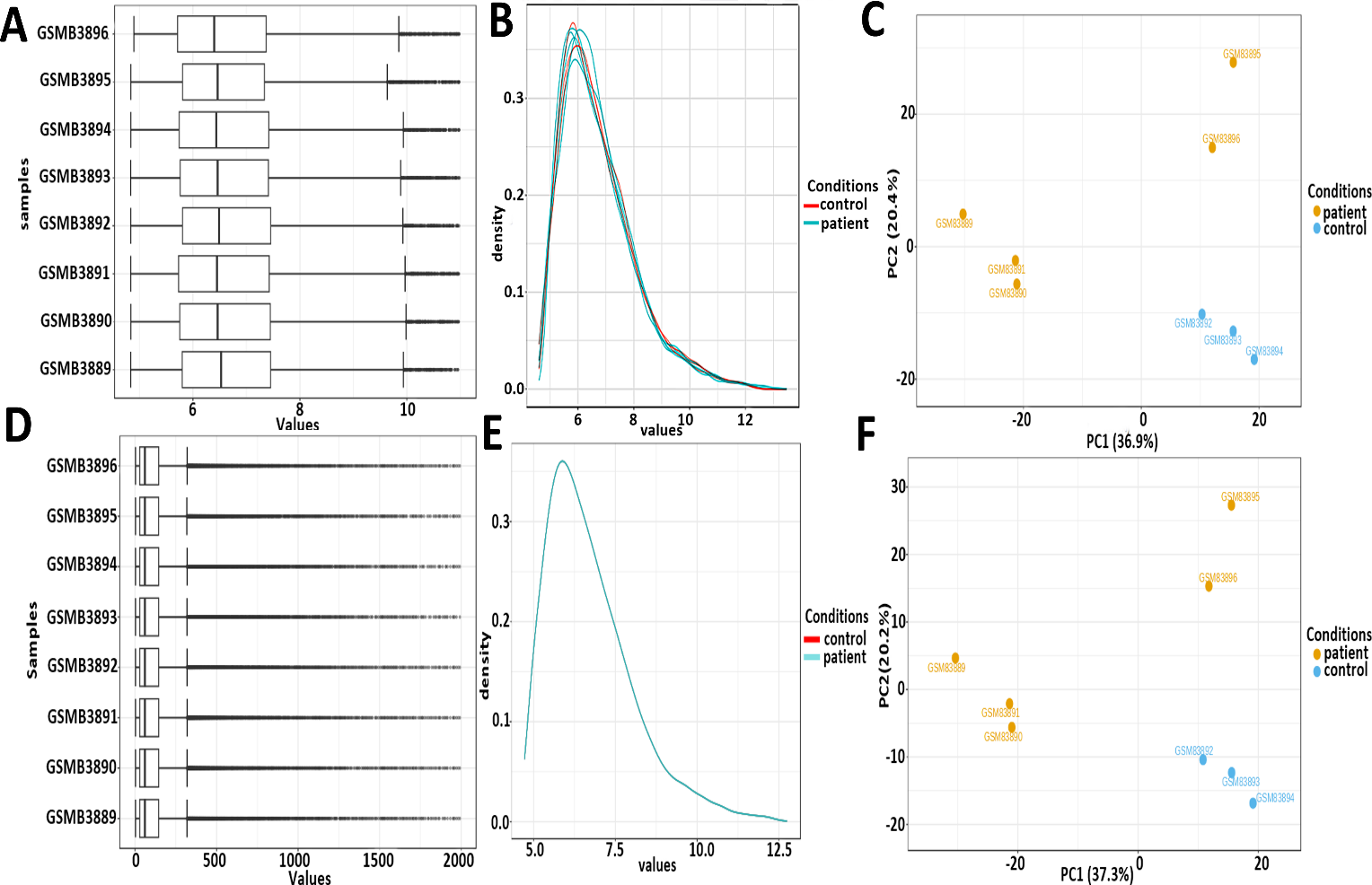


Figure S1. Prior to normalization, the box plot, density plot, and mean expression plot are shown in (A), (B), and (C); following normalization, the same plots are shown in (D), (E), and (F). Using the Network Analyst Tool, variance-stabilizing normalization successfully removed noise from the data. The next methods used to analyze the data were Principal Component Analysis and the Quality Control Examination.

**
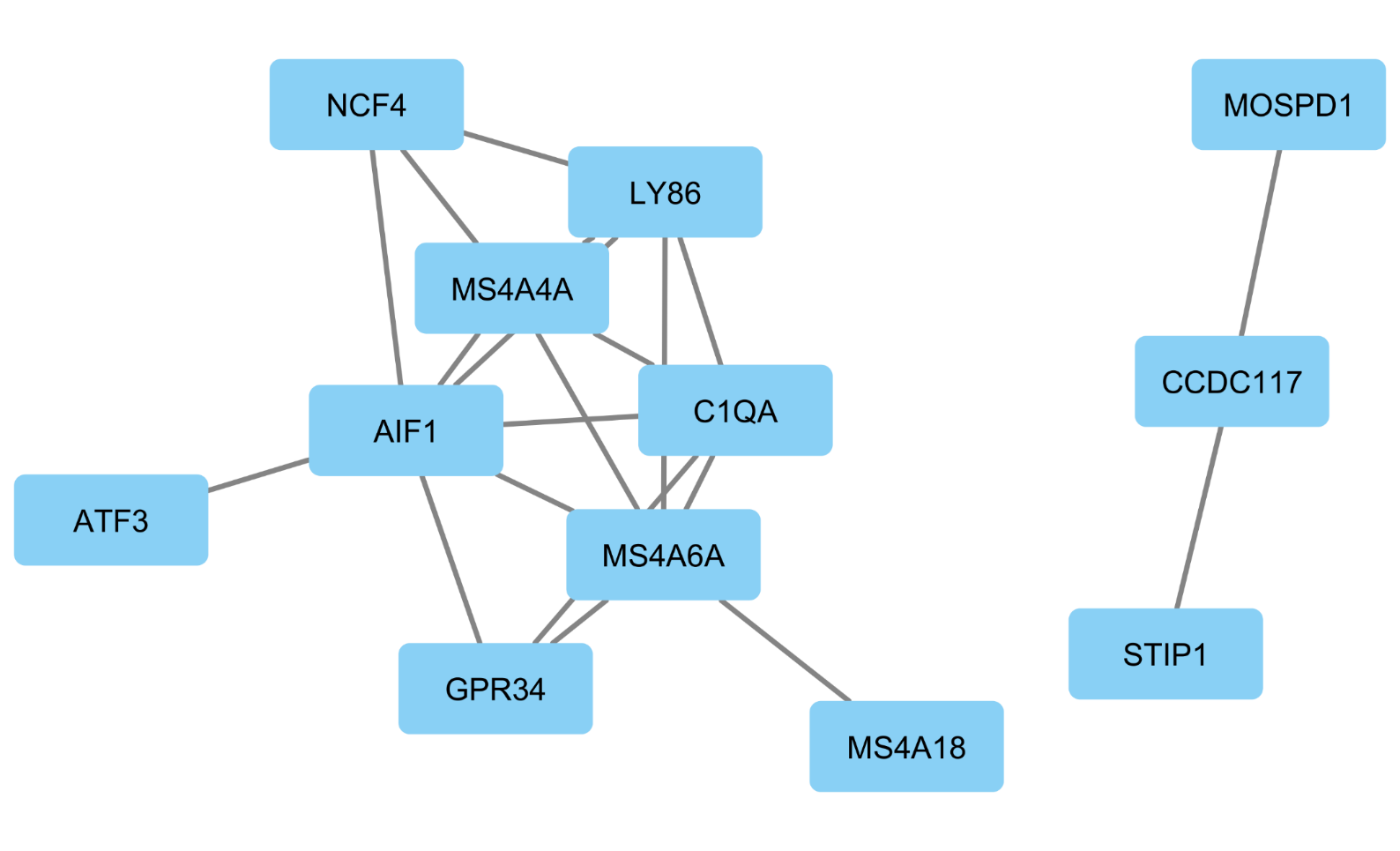
**

**Figure S2.** The network comprised 12 edges and 20 nodes. The topological measurements of the investigation indicated strong links throughout the network, with a mean node value of 1 and a mean local clustering coefficient of 0.186. The PPI data of the differentially expressed genes (DEGs) were represented using Cytoscape software and the STRING database.


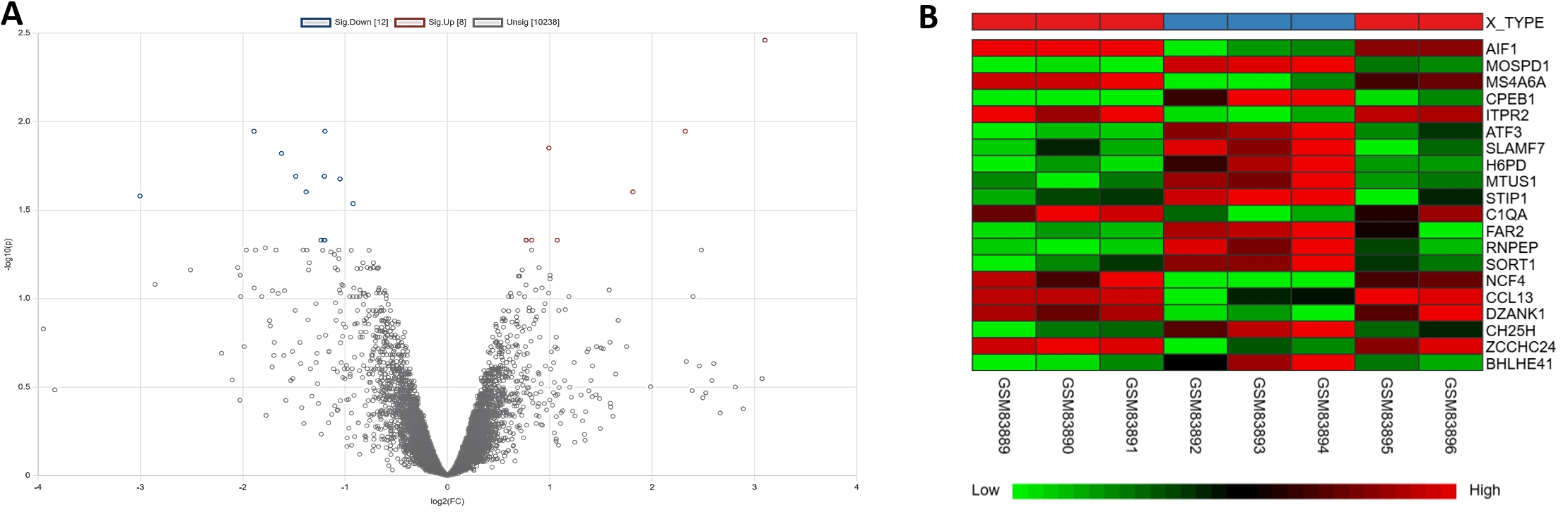


**Figure S3.** (A) Presenting the level of expression distribution of each gene on a volcano plot, with non-significant genes in the specimens shown in grey according to a False Discovery Rate (FDR) threshold above 2, upregulated genes marked in red, and downregulated genes in blue. (B) Employing a heat map to illustrate gene expression levels across all samples. The legend illustrates the distribution of expression, ranging from low (green) to high (red).


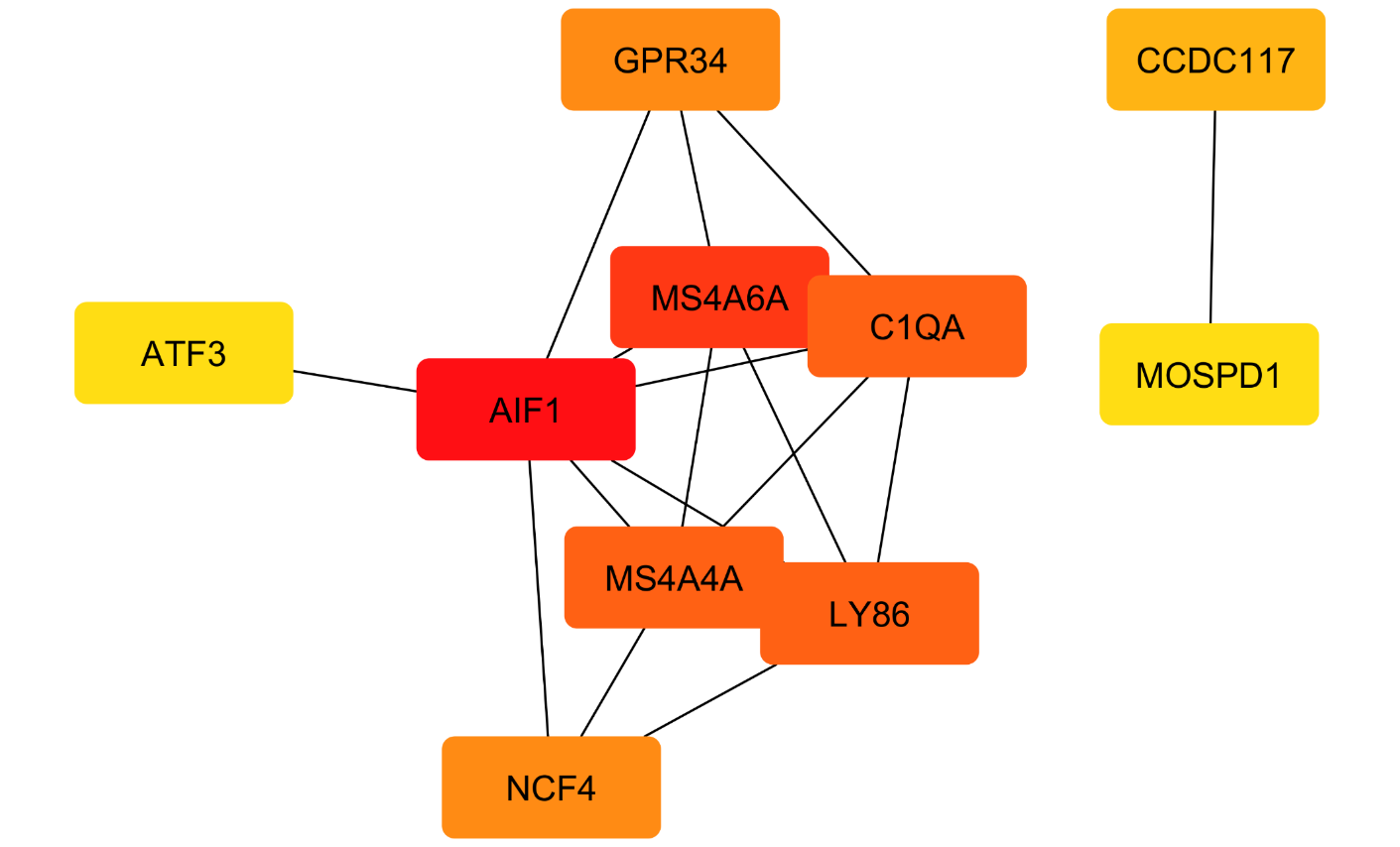


**Figure S4.** All identified hub genes are presented with their shortest path interactions, with nodes colour-coded according to their degree scores.


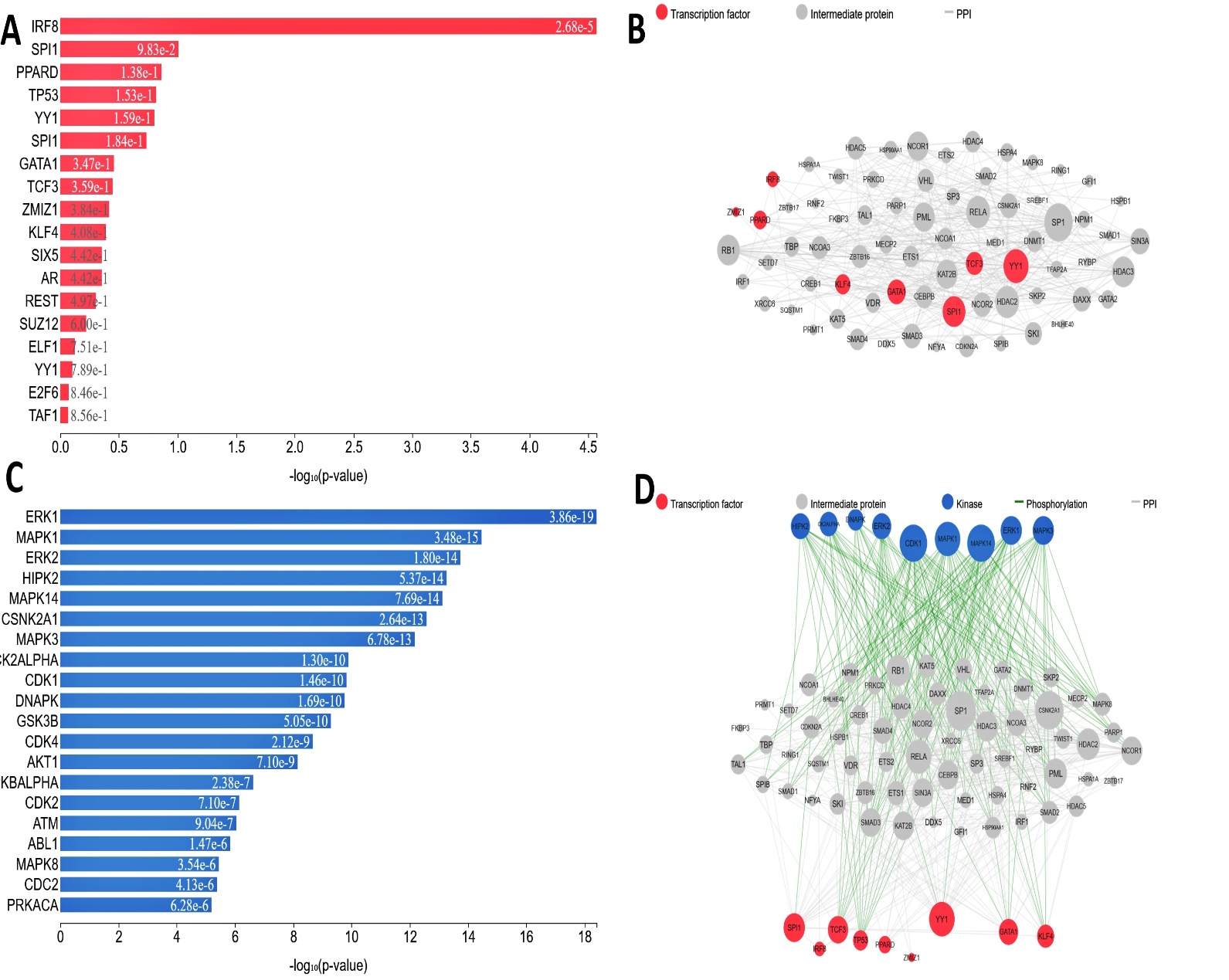


**Figure S5.** (A) Bar graph illustrates hypergeometric p-values and scores for predicted transcription factors associated with differentially expressed genes (DEGs). (B) The transcript interaction network is shown by a sphere-and-stick model, with node size reflecting connectedness. Grey nodes denote connecting peptides, whereas pink nodes signify transcription factors. (C) Kinase ranking based on hypergeometric p-values from X2K Web study. (D) The PPI networks for the principal kinases linked to differential gene expression are illustrated.

**Table S1.** The STRING protein-protein interaction database for the GSE25496 dataset identifies the top 10 genes derived from the DEGs network analysis. The table also includes the fold changes and adjusted values for each gene.

| **Rank** | **Gene name** | **Score** | **logFC** | **Adjusted P-value** |
| --- | --- | --- | --- | --- |
| 1 | AIF1 | 7 | 3.1025 | 0.003463 |
| 2 | MS4A6A | 6 | 2.3239 | 0.011329 |
| 3 | C1QA | 5 | 1.8122 | 0.024925 |
| 3 | MS4A4A | 5 | 1.0617 | 0.63858 |
| 3 | LY86 | 5 | 0.55715 | 0.50275 |
| 6 | NCF4 | 3 | 0.82345 | 0.046733 |
| 6 | GPR34 | 3 | - 0.11991 | 0.92912 |
| 8 | CCDC117 | 2 | 0.024423 | 0.98629 |
| 9 | MOSPD1 | 1 | - 1.1971 | 0.011329 |
| 9 | ATF3 | 1 | - 1.6217 | 0.01511 |

**Table S2.** Transcription factor identified during the network analysis with hypergeometric p-value and Z score.

| **Rank** | **Transcription Factor** | **Hypergeometric p-value** | **Z-score** | **Combined score** |
| --- | --- | --- | --- | --- |
| 1 | UBTF | 0.003367 | 0.00 | 0.00 |
| 2 | NFIC | 0.01101 | 0.00 | 0.00 |
| 3 | PPARG | 0.02451 | 0.00 | 0.00 |
| 4 | AR | 0.05003 | 0.00 | 0.00 |
| 5 | IRF8 | 0.05492 | 0.00 | 0.00 |
| 6 | RCOR1 | 0.06492 | 0.00 | 0.00 |
| 7 | SMC3 | 0.06947 | 0.00 | 0.00 |
| 8 | SUZ12 | 0.0703 | 0.00 | 0.00 |
| 9 | ESR1 | 0.08308 | 0.00 | 0.00 |
| 10 | SPI1 | 0.09806 | 0.00 | 0.00 |
| 11 | SMAD4 | 0.106 | 0.00 | 0.00 |
| 12 | TCF3 | 0.1164 | 0.00 | 0.00 |
| 13 | ZMIZ1 | 0.1503 | 0.00 | 0.00 |
| 14 | SP1 | 0.1739 | 0.00 | 0.00 |
| 15 | HNF4A | 0.1984 | 0.00 | 0.00 |
| 16 | NFE2L2 | 0.2065 | 0.00 | 0.00 |
| 17 | RUNX1 | 0.2071 | 0.00 | 0.00 |
| 18 | SOX2 | 0.2168 | 0.00 | 0.00 |
| 19 | PPARD | 0.2206 | 0.00 | 0.00 |
| 20 | CBX3 | 0.2237 | 0.00 | 0.00 |
| 21 | SPI1 | 0.2255 | 0.00 | 0.00 |
| 22 | GATA1 | 0.2379 | 0.00 | 0.00 |
| 23 | RFX5 | 0.2487 | 0.00 | 0.00 |
| 24 | TP53 | 0.2594 | 0.00 | 0.00 |
| 25 | TP63 | 0.2609 | 0.00 | 0.00 |
| 26 | ATF3 | 0.2992 | 0.00 | 0.00 |
| 27 | CTCF | 0.3129 | 0.00 | 0.00 |
| 28 | FOS | 0.3144 | 0.00 | 0.00 |
| 29 | KLF4 | 0.3636 | 0.00 | 0.00 |
| 30 | ETS1 | 0.3849 | 0.00 | 0.00 |
| 31 | HDAC2 | 0.4202 | 0.00 | 0.00 |
| 32 | STAT3 | 0.4238 | 0.00 | 0.00 |
| 33 | SIN3A | 0.4653 | 0.00 | 0.00 |
| 34 | E2F1 | 0.499 | 0.00 | 0.00 |
| 35 | NELFE | 0.5177 | 0.00 | 0.00 |
| 36 | TCF7L2 | 0.5417 | 0.00 | 0.00 |
| 37 | NFYA | 0.5527 | 0.00 | 0.00 |
| 38 | NANOG | 0.5529 | 0.00 | 0.00 |
| 39 | RAD21 | 0.5551 | 0.00 | 0.00 |
| 40 | POU5F1 | 0.5568 | 0.00 | 0.00 |
| 41 | PBX3 | 0.5577 | 0.00 | 0.00 |
| 42 | SP2 | 0.5994 | 0.00 | 0.00 |
| 43 | IRF3 | 0.6124 | 0.00 | 0.00 |
| 44 | NFYB | 0.6168 | 0.00 | 0.00 |
| 45 | EGR1 | 0.6259 | 0.00 | 0.00 |
| 46 | FOXA2 | 0.627 | 0.00 | 0.00 |
| 47 | EZH2 | 0.652 | 0.00 | 0.00 |
| 48 | STAT3 | 0.6615 | 0.00 | 0.00 |
| 49 | ZNF384 | 0.6652 | 0.00 | 0.00 |
| 50 | SALL4 | 0.6701 | 0.00 | 0.00 |
| 51 | ZBTB7A | 0.6825 | 0.00 | 0.00 |
| 52 | GATA2 | 0.6954 | 0.00 | 0.00 |
| 53 | USF2 | 0.8068 | 0.00 | 0.00 |
| 54 | TCF3 | 0.8253 | 0.00 | 0.00 |
| 55 | USF1 | 0.827 | 0.00 | 0.00 |
| 56 | CREB1 | 0.8346 | 0.00 | 0.00 |
| 57 | MYC | 0.8349 | 0.00 | 0.00 |
| 58 | TAF7 | 0.8668 | 0.00 | 0.00 |
| 59 | PML | 0.8821 | 0.00 | 0.00 |
| 60 | E2F4 | 0.8937 | 0.00 | 0.00 |
| 61 | CEBPD | 0.9016 | 0.00 | 0.00 |
| 62 | NRF1 | 0.9403 | 0.00 | 0.00 |
| 63 | TAF1 | 0.9622 | 0.00 | 0.00 |
| 64 | REST | 0.9837 | 0.00 | 0.00 |
| 65 | ATF2 | 0.984 | 0.00 | 0.00 |
| 66 | ELF1 | 0.9879 | 0.00 | 0.00 |
| 67 | MAX | 0.9912 | 0.00 | 0.00 |
| 68 | BRCA1 | 0.9941 | 0.00 | 0.00 |
| 69 | E2F6 | 0.9945 | 0.00 | 0.00 |
| 70 | YY1 | 0.999 | 0.00 | 0.00 |
| 71 | CREB1 | 0.9994 | 0.00 | 0.00 |

**Table S3.** Gene involved in protein kinase retrieved during network analysis with Hypergeometric p-value and Z score.

| **Rank** | **Protein Kinase** | **Hypergeometric p-value** | **Z-score** | **Combined score** |
| --- | --- | --- | --- | --- |
| 1 | ERK2 | 2.227e-21 | 0.00 | 0.00 |
| 2 | MAPK1 | 3.914e-19 | 0.00 | 0.00 |
| 3 | CSNK2A1 | 1.464e-18 | 0.00 | 0.00 |
| 4 | MAPK14 | 1.514e-18 | 0.00 | 0.00 |
| 5 | ERK1 | 1.676e-18 | 0.00 | 0.00 |
| 6 | GSK3B | 2.800e-18 | 0.00 | 0.00 |
| 7 | CDK1 | 8.316e-17 | 0.00 | 0.00 |
| 8 | MAPK3 | 8.319e-17 | 0.00 | 0.00 |
| 9 | CDK4 | 6.596e-12 | 0.00 | 0.00 |
| 10 | HIPK2 | 7.297e-12 | 0.00 | 0.00 |
| 11 | GSK3BETA | 1.595e-11 | 0.00 | 0.00 |
| 12 | MAPK8 | 1.442e-10 | 0.00 | 0.00 |
| 13 | AKT1 | 4.543e-10 | 0.00 | 0.00 |
| 14 | JNK1 | 6.263e-10 | 0.00 | 0.00 |
| 15 | CK2ALPHA | 1.867e-9 | 0.00 | 0.00 |
| 16 | CDK2 | 3.411e-9 | 0.00 | 0.00 |
| 17 | GSK | 3.679e-9 | 0.00 | 0.00 |
| 18 | ERBB2 | 7.168e-9 | 0.00 | 0.00 |
| 19 | ABL1 | 8.443e-9 | 0.00 | 0.00 |
| 20 | SRC | 1.062e-8 | 0.00 | 0.00 |
| 21 | CHUK | 6.615e-8 | 0.00 | 0.00 |
| 22 | JNK2 | 1.473e-7 | 0.00 | 0.00 |
| 23 | PKBALPHA | 1.662e-7 | 0.00 | 0.00 |
| 24 | EGFR | 2.812e-7 | 0.00 | 0.00 |
| 25 | PRKCA | 5.697e-7 | 0.00 | 0.00 |
| 26 | DNAPK | 5.751e-7 | 0.00 | 0.00 |
| 27 | CDC2 | 7.803e-7 | 0.00 | 0.00 |
| 28 | IKK | 0.000001656 | 0.00 | 0.00 |
| 29 | MAP2K1 | 0.000002377 | 0.00 | 0.00 |
| 30 | GSK3ALPHA | 0.000002938 | 0.00 | 0.00 |
| 31 | PRKACA | 0.000006536 | 0.00 | 0.00 |
| 32 | IKKBETA | 0.00002033 | 0.00 | 0.00 |
| 33 | RAF1 | 0.0000212 | 0.00 | 0.00 |
| 34 | RPS6KA3 | 0.00002318 | 0.00 | 0.00 |
| 35 | TGFBR2 | 0.00002349 | 0.00 | 0.00 |
| 36 | GSK3A | 0.0000313 | 0.00 | 0.00 |
| 37 | PRKCZ | 0.00005492 | 0.00 | 0.00 |
| 38 | MAPK11 | 0.00008065 | 0.00 | 0.00 |
| 39 | PKD1 | 0.00008732 | 0.00 | 0.00 |
| 40 | PRKCB | 0.0001255 | 0.00 | 0.00 |
| 41 | TYK2 | 0.0002189 | 0.00 | 0.00 |
| 42 | PKBBETA | 0.0002493 | 0.00 | 0.00 |
| 43 | PRKCD | 0.0002525 | 0.00 | 0.00 |
| 44 | PRKDC | 0.0002868 | 0.00 | 0.00 |
| 45 | INSR | 0.0003063 | 0.00 | 0.00 |
| 46 | RPS6KA1 | 0.0003481 | 0.00 | 0.00 |
| 47 | PKCEPSILON | 0.0003707 | 0.00 | 0.00 |
| 48 | ATM | 0.0004624 | 0.00 | 0.00 |
| 49 | CDK3 | 0.0005531 | 0.00 | 0.00 |
| 50 | IKBKB | 0.0005947 | 0.00 | 0.00 |
| 51 | CSNK1E | 0.0007598 | 0.00 | 0.00 |
| 52 | CDK9 | 0.0007603 | 0.00 | 0.00 |
| 53 | ACTR2B | 0.0007837 | 0.00 | 0.00 |
| 54 | PKC | 0.0009329 | 0.00 | 0.00 |
| 55 | ALK | 0.001017 | 0.00 | 0.00 |
| 56 | CSNK2A2 | 0.001129 | 0.00 | 0.00 |
| 57 | BTK | 0.00113 | 0.00 | 0.00 |
| 58 | CDK7 | 0.001812 | 0.00 | 0.00 |
| 59 | TBK1 | 0.002248 | 0.00 | 0.00 |
| 60 | MAP2K3 | 0.002481 | 0.00 | 0.00 |
| 61 | AURORAA | 0.002623 | 0.00 | 0.00 |
| 62 | IKKALPHA | 0.00279 | 0.00 | 0.00 |
| 63 | CHEK1 | 0.003228 | 0.00 | 0.00 |
| 64 | JAK2 | 0.003255 | 0.00 | 0.00 |
| 65 | PIM1 | 0.003472 | 0.00 | 0.00 |
| 66 | MAPK9 | 0.003669 | 0.00 | 0.00 |
| 67 | IGF1R | 0.003698 | 0.00 | 0.00 |
| 68 | RSK | 0.0041 | 0.00 | 0.00 |
| 69 | PKBGAMMA | 0.004636 | 0.00 | 0.00 |
| 70 | CDK8 | 0.004636 | 0.00 | 0.00 |
| 71 | UHMK1 | 0.005075 | 0.00 | 0.00 |
| 72 | MAPK7 | 0.005075 | 0.00 | 0.00 |
| 73 | PKC | 0.007358 | 0.00 | 0.00 |
| 74 | CAMKIV | 0.007358 | 0.00 | 0.00 |
| 75 | PRKAA1 | 0.007408 | 0.00 | 0.00 |
| 76 | JNK3 | 0.008634 | 0.00 | 0.00 |
| 77 | MTOR | 0.009201 | 0.00 | 0.00 |
| 78 | PKAALPHA | 0.009526 | 0.00 | 0.00 |
| 79 | TRKB | 0.01 | 0.00 | 0.00 |
| 80 | PDGFRALPHA | 0.01 | 0.00 | 0.00 |
| 81 | BRD2 | 0.01 | 0.00 | 0.00 |
| 82 | ADRBK1 | 0.01 | 0.00 | 0.00 |
| 83 | PAK1 | 0.01127 | 0.00 | 0.00 |
| 84 | LCK | 0.01151 | 0.00 | 0.00 |
| 85 | RPS6KA5 | 0.01233 | 0.00 | 0.00 |
| 86 | IKBKE | 0.01356 | 0.00 | 0.00 |
| 87 | PKCIOTA | 0.01474 | 0.00 | 0.00 |
| 88 | PKCALPHA | 0.01681 | 0.00 | 0.00 |
| 89 | CK1ALPHA | 0.01709 | 0.00 | 0.00 |
| 90 | PRKCI | 0.01835 | 0.00 | 0.00 |
| 91 | PKA | 0.02398 | 0.00 | 0.00 |
| 92 | CSNK2B | 0.02398 | 0.00 | 0.00 |
| 93 | CAMKIIBETA | 0.0246 | 0.00 | 0.00 |
| 94 | MAPK10 | 0.02574 | 0.00 | 0.00 |
| 95 | PIM3 | 0.02608 | 0.00 | 0.00 |
| 96 | CAM | 0.02608 | 0.00 | 0.00 |
| 97 | ABL | 0.0281 | 0.00 | 0.00 |
| 98 | MAPK6 | 0.02825 | 0.00 | 0.00 |
| 99 | PKAGAMMA | 0.02933 | 0.00 | 0.00 |
| 100 | CDK16 | 0.03049 | 0.00 | 0.00 |
| 101 | MERTK | 0.03049 | 0.00 | 0.00 |
| 102 | AKT2 | 0.03186 | 0.00 | 0.00 |
| 103 | CAMKIIALPHA | 0.03186 | 0.00 | 0.00 |
| 104 | LYN | 0.03275 | 0.00 | 0.00 |
| 105 | RPS6KA4 | 0.0328 | 0.00 | 0.00 |
| 106 | PLK1 | 0.03706 | 0.00 | 0.00 |
| 107 | MOK | 0.03762 | 0.00 | 0.00 |
| 108 | MELK | 0.04012 | 0.00 | 0.00 |
| 109 | CK2 | 0.04159 | 0.00 | 0.00 |
| 110 | IRAK1 | 0.04268 | 0.00 | 0.00 |
| 111 | RPS6KB1 | 0.04309 | 0.00 | 0.00 |
| 112 | JAK3 | 0.04531 | 0.00 | 0.00 |
| 113 | CDK6 | 0.04531 | 0.00 | 0.00 |
| 114 | PDHK1 | 0.04617 | 0.00 | 0.00 |
| 115 | SYK | 0.04775 | 0.00 | 0.00 |
| 116 | PRKACB | 0.04799 | 0.00 | 0.00 |
| 117 | FLT1 | 0.04799 | 0.00 | 0.00 |
| 118 | LATS2 | 0.04799 | 0.00 | 0.00 |
| 119 | AXL | 0.05073 | 0.00 | 0.00 |
| 120 | FER | 0.05073 | 0.00 | 0.00 |
| 121 | PRKD1 | 0.05099 | 0.00 | 0.00 |
| 122 | CAMK2A | 0.0535 | 0.00 | 0.00 |
| 123 | BARK1 | 0.05778 | 0.00 | 0.00 |
| 124 | CSK | 0.05927 | 0.00 | 0.00 |
| 125 | PRKCE | 0.06314 | 0.00 | 0.00 |
| 126 | CAM | 0.06426 | 0.00 | 0.00 |
| 127 | PKM | 0.06426 | 0.00 | 0.00 |
| 128 | FYN | 0.06678 | 0.00 | 0.00 |
| 129 | RET | 0.06827 | 0.00 | 0.00 |
| 130 | AURKA | 0.06872 | 0.00 | 0.00 |
| 131 | MAPK12 | 0.07256 | 0.00 | 0.00 |
| 132 | DYRK2 | 0.0745 | 0.00 | 0.00 |
| 133 | PKC | 0.07457 | 0.00 | 0.00 |
| 134 | NIK | 0.07457 | 0.00 | 0.00 |
| 135 | SLK | 0.07768 | 0.00 | 0.00 |
| 136 | PRKAA2 | 0.07768 | 0.00 | 0.00 |
| 137 | PDGFRBETA | 0.07768 | 0.00 | 0.00 |
| 138 | HCK | 0.07768 | 0.00 | 0.00 |
| 139 | KIT | 0.07768 | 0.00 | 0.00 |
| 140 | MAP2K2 | 0.08091 | 0.00 | 0.00 |
| 141 | DYRK1A | 0.08091 | 0.00 | 0.00 |
| 142 | ABL2 | 0.08091 | 0.00 | 0.00 |
| 143 | PKR | 0.08417 | 0.00 | 0.00 |
| 144 | RSK3 | 0.08476 | 0.00 | 0.00 |
| 145 | MET | 0.08748 | 0.00 | 0.00 |
| 146 | PRKD2 | 0.0942 | 0.00 | 0.00 |
| 147 | DNA | 0.09484 | 0.00 | 0.00 |
| 148 | MAP3K7 | 0.09976 | 0.00 | 0.00 |
| 149 | PAK4 | 0.1046 | 0.00 | 0.00 |
| 150 | TRRAP | 0.1048 | 0.00 | 0.00 |
| 151 | RSK | 0.1048 | 0.00 | 0.00 |
| 152 | ZAK | 0.1048 | 0.00 | 0.00 |
| 153 | CHEK2 | 0.1081 | 0.00 | 0.00 |
| 154 | PKCTHETA | 0.1152 | 0.00 | 0.00 |
| 155 | ERBB3 | 0.141 | 0.00 | 0.00 |
| 156 | P70S6K | 0.1436 | 0.00 | 0.00 |
| 157 | TGFBR1 | 0.1486 | 0.00 | 0.00 |
| 158 | CK2A2 | 0.1563 | 0.00 | 0.00 |
| 159 | ATR | 0.1586 | 0.00 | 0.00 |
| 160 | CDK5 | 0.1587 | 0.00 | 0.00 |
| 161 | PRKCQ | 0.1719 | 0.00 | 0.00 |
| 162 | TRKA | 0.1808 | 0.00 | 0.00 |
| 163 | CDC42BPG | 0.1808 | 0.00 | 0.00 |
| 164 | RIPK4 | 0.1898 | 0.00 | 0.00 |
| 165 | TNK1 | 0.1898 | 0.00 | 0.00 |
| 166 | CDC42BPB | 0.1898 | 0.00 | 0.00 |
| 167 | RPS6KA6 | 0.1898 | 0.00 | 0.00 |
| 168 | TRIO | 0.1988 | 0.00 | 0.00 |
| 169 | CDK12 | 0.1988 | 0.00 | 0.00 |
| 170 | RNASEL | 0.1988 | 0.00 | 0.00 |
| 171 | ULK4 | 0.1988 | 0.00 | 0.00 |
| 172 | MINK1 | 0.1988 | 0.00 | 0.00 |
| 173 | PKCGAMMA | 0.1997 | 0.00 | 0.00 |
| 174 | MAPK13 | 0.2037 | 0.00 | 0.00 |
| 175 | DMPK | 0.2076 | 0.00 | 0.00 |
| 176 | PKN3 | 0.2076 | 0.00 | 0.00 |
| 177 | HIPK3 | 0.2076 | 0.00 | 0.00 |
| 178 | TAOK2 | 0.2076 | 0.00 | 0.00 |
| 179 | PRPF4B | 0.2076 | 0.00 | 0.00 |
| 180 | PKMYT1 | 0.2076 | 0.00 | 0.00 |
| 181 | NTRK3 | 0.2076 | 0.00 | 0.00 |
| 182 | MST1R | 0.2076 | 0.00 | 0.00 |
| 183 | MARK4 | 0.2076 | 0.00 | 0.00 |
| 184 | SCYL1 | 0.2076 | 0.00 | 0.00 |
| 185 | PAK2 | 0.2118 | 0.00 | 0.00 |
| 186 | AURORAB | 0.2158 | 0.00 | 0.00 |
| 187 | PKN2 | 0.2163 | 0.00 | 0.00 |
| 188 | PIK3CG | 0.2163 | 0.00 | 0.00 |
| 189 | TLK2 | 0.2163 | 0.00 | 0.00 |
| 190 | TRIB3 | 0.2163 | 0.00 | 0.00 |
| 191 | MAPK15 | 0.2163 | 0.00 | 0.00 |
| 192 | HIPK1 | 0.225 | 0.00 | 0.00 |
| 193 | MAP3K6 | 0.225 | 0.00 | 0.00 |
| 194 | NEK9 | 0.225 | 0.00 | 0.00 |
| 195 | STK24 | 0.225 | 0.00 | 0.00 |
| 196 | STK26 | 0.225 | 0.00 | 0.00 |
| 197 | AMPKA2 | 0.225 | 0.00 | 0.00 |
| 198 | SIK1 | 0.225 | 0.00 | 0.00 |
| 199 | PIK3R4 | 0.225 | 0.00 | 0.00 |
| 200 | MAP4K4 | 0.225 | 0.00 | 0.00 |
| 201 | CSNK1A1 | 0.2321 | 0.00 | 0.00 |
| 202 | FRK | 0.2335 | 0.00 | 0.00 |
| 203 | CSNK1G1 | 0.2335 | 0.00 | 0.00 |
| 204 | EPHB4 | 0.2335 | 0.00 | 0.00 |
| 205 | DYRK3 | 0.2335 | 0.00 | 0.00 |
| 206 | OXSR1 | 0.2335 | 0.00 | 0.00 |
| 207 | PBK | 0.242 | 0.00 | 0.00 |
| 208 | YES | 0.242 | 0.00 | 0.00 |
| 209 | GRK6 | 0.242 | 0.00 | 0.00 |
| 210 | MKNK2 | 0.242 | 0.00 | 0.00 |
| 211 | PIM2 | 0.2504 | 0.00 | 0.00 |
| 212 | EPHB3 | 0.2504 | 0.00 | 0.00 |
| 213 | EPHA8 | 0.2504 | 0.00 | 0.00 |
| 214 | SIK2 | 0.2504 | 0.00 | 0.00 |
| 215 | VRK1 | 0.2586 | 0.00 | 0.00 |
| 216 | MOS | 0.2586 | 0.00 | 0.00 |
| 217 | MKNK1 | 0.2586 | 0.00 | 0.00 |
| 218 | CAMKIIGAMMA | 0.2668 | 0.00 | 0.00 |
| 219 | CLK2 | 0.2668 | 0.00 | 0.00 |
| 220 | TNK2 | 0.2749 | 0.00 | 0.00 |
| 221 | DYRK1B | 0.2829 | 0.00 | 0.00 |
| 222 | LKB1 | 0.2829 | 0.00 | 0.00 |
| 223 | PTK6 | 0.2829 | 0.00 | 0.00 |
| 224 | CAMK4 | 0.2829 | 0.00 | 0.00 |
| 225 | GRK2 | 0.2829 | 0.00 | 0.00 |
| 226 | PRKCH | 0.2909 | 0.00 | 0.00 |
| 227 | JAK1 | 0.2909 | 0.00 | 0.00 |
| 228 | CAMK2D | 0.2909 | 0.00 | 0.00 |
| 229 | TEC | 0.2909 | 0.00 | 0.00 |
| 230 | PKN1 | 0.2987 | 0.00 | 0.00 |
| 231 | NEK6 | 0.2987 | 0.00 | 0.00 |
| 232 | PLK2 | 0.3064 | 0.00 | 0.00 |
| 233 | EIF2AK2 | 0.3064 | 0.00 | 0.00 |
| 234 | EPHB1 | 0.3141 | 0.00 | 0.00 |
| 235 | BMPR1B | 0.3141 | 0.00 | 0.00 |
| 236 | ZAP70 | 0.3141 | 0.00 | 0.00 |
| 237 | DAPK3 | 0.3217 | 0.00 | 0.00 |
| 238 | PDGFRB | 0.3217 | 0.00 | 0.00 |
| 239 | PAK6 | 0.3292 | 0.00 | 0.00 |
| 240 | PKG1CGKI | 0.3366 | 0.00 | 0.00 |
| 241 | ITK | 0.3439 | 0.00 | 0.00 |
| 242 | MARK2 | 0.3512 | 0.00 | 0.00 |
| 243 | RIPK3 | 0.3541 | 0.00 | 0.00 |
| 244 | PLK3 | 0.3584 | 0.00 | 0.00 |
| 245 | BLK | 0.3584 | 0.00 | 0.00 |
| 246 | EPHA2 | 0.3794 | 0.00 | 0.00 |
| 247 | RIPK1 | 0.3794 | 0.00 | 0.00 |
| 248 | MAP2K6 | 0.3931 | 0.00 | 0.00 |
| 249 | CSNK1D | 0.3998 | 0.00 | 0.00 |
| 250 | NLK | 0.3998 | 0.00 | 0.00 |
| 251 | ROCK2 | 0.4064 | 0.00 | 0.00 |
| 252 | PAK3 | 0.4064 | 0.00 | 0.00 |
| 253 | MAP3K14 | 0.4207 | 0.00 | 0.00 |
| 254 | KDR | 0.4323 | 0.00 | 0.00 |
| 255 | PKABETA | 0.4323 | 0.00 | 0.00 |
| 256 | MAP2K4 | 0.4386 | 0.00 | 0.00 |
| 257 | MAP3K1 | 0.4979 | 0.00 | 0.00 |
| 258 | MAP3K8 | 0.5197 | 0.00 | 0.00 |
| 259 | SGK1 | 0.5197 | 0.00 | 0.00 |
| 260 | MAPKAPK2 | 0.5303 | 0.00 | 0.00 |
| 261 | TRIM24 | 0.5407 | 0.00 | 0.00 |
| 262 | ROCK1 | 0.5558 | 0.00 | 0.00 |
| 263 | PKC | 0.5938 | 0.00 | 0.00 |
| 264 | MAP3K3 | 0.6172 | 0.00 | 0.00 |
| 265 | TTK | 0.686 | 0.00 | 0.00 |
| 266 | AURKB | 0.8231 | 0.00 | 0.00 |

**Table S4.** Lipinski Rule of five calculated for the selected inhibitors using SWISS-ADME

| **Molecule** | **MW** | **Heavy atoms** | **Aromatic heavy atoms** | **Fraction Csp3** | **Rotatable bonds** | **H-bond acceptors** | **H-bond donors** | **TPSA** | **Water** | **GI absorption** | **BBB** | **CYP2D6 inhibitor** | **Bioavailability Score** | **PAINS** |
| --- | --- | --- | --- | --- | --- | --- | --- | --- | --- | --- | --- | --- | --- | --- |
| AMG-131 | 514.21 | 31 | 22 | 0 | 5 | 4 | 1 | 76.67 | Poorly soluble | Low | No | Yes | 0.17 | 0 |
| Elafibranor | 384.49 | 27 | 12 | 0.27 | 7 | 4 | 1 | 88.9 | Moderately soluble | High | No | No | 0.56 | 0 |
| Rosiglitazone (S) | 357.43 | 25 | 12 | 0.28 | 7 | 4 | 1 | 96.83 | Soluble | High | No | Yes | 0.55 | 0 |
| Samarium Sm-153 Lexidronam | 436.12 | 24 | 0 | 1 | 11 | 14 | 8 | 275.84 | Highly soluble | Low | No | No | 0.11 | 0 |
| Clemastine | 343.89 | 24 | 12 | 0.43 | 6 | 2 | 0 | 12.47 | Moderately soluble | High | Yes | Yes | 0.55 | 0 |

**Table S5.** Molecular docking results for the receptors (1FM6, 2g2b and 1udw) against the FDA approved inhibitors with positive and negative control.

| S.no | Chemical name | IUPAC name | Docked with (**1FM6- receptor**) Binding affinities (kcal/mol) | Docked with **(2g2b- receptor**) Binding affinities (kcal/mol) | Docked with **(1udw- receptor**) Binding affinities (kcal/mol) |
| --- | --- | --- | --- | --- | --- |
| 1 | AMG-131 | [ 2,4-dichloro-N-(3,5-dichloro-4-quinolin-3-yloxyphenyl)benzenesulfonamide] | -7.4 | -6.9 | -7.1 |
| 2 | Elafibranor | [ 2-[2,6-dimethyl-4-[(E)-3-(4-methyl sulfanyl phenyl)-3-oxoprop-1-enyl]phenoxy]-2-methyl propanoic acid] | -7.2 | -5.8 | -5.6 |
| 3 | Positive Control- Rosiglitazone (S) | 5-[[4-[2-[methyl(pyridin-2-yl)amino]ethoxy]phenyl]methyl]-1,3-thiazolidine-2,4-dione | -7.5 | -6.1 | -6.1 |
| 4 | Samarium Sm-153 Lexidronam | [2-[bis[[hydroxy(oxido)phosphoryl]methyl]amino]ethyl-(phosphonomethyl)amino]methyl-hydroxyphosphinate;samarium-153(3+) | -5.2 | -4.6 | -5.1 |
| 5 | Clemastine | (2*R*)-2-[2-[(1*R*)-1-(4-chlorophenyl)-1-phenylethoxy]ethyl]-1-methylpyrrolidine | -6.1 | -6.9 | -5.6 |
| 6 | Negative control, Acetaminophen | *N*-(4-hydroxyphenyl)acetamide | -5.2 | -4.5 | -4.3 |
