# Supplementary material for: System biology and network-based approach to identify the therapeutic signatures and potential inhibitors against polycystic lipomembranous osteodysplasia with Sclerosing Leukoencephalopathy
Source: PLoS One. 2026 Feb 20;21(2):e0343274. doi: 10.1371/journal.pone.0343274 (PMC12923142; doi:10.1371/journal.pone.0343274)
Supplement: Attachment — Submitted filename: PONE-D-25-34800_Comments.docx [file pone.0343274.s002.docx]

The authors have conducted a good work addressing a relevant scientific problem and have presented their analyses clearly. A few constructive suggestions have been provided to enhance clarity and strengthen the overall quality of the manuscript.

1. The study uses very small sample sizes, which limits statistical confidence. The authors should acknowledge the limitation clearly in the Discussion and Conclusion.
2. The authors should strengthen the rationale for selecting PPARG as the primary hub gene. Ranking by degree score alone is not sufficient; additional biological justification and supporting literature linking PPARG specifically to PLOSL pathology should be provided.
3. Survival analysis appears inappropriate because no PLOSL clinical survival dataset exists. The analysis should be removed or explicitly clarified as exploratory and non-disease-specific.
4. The authors should reduce repetitive content, particularly in the Introduction and Discussion, to improve clarity and maintain a concise narrative.
5. The PCA and heatmap figures require improved resolution and clearer labeling to allow interpretability.
6. Figures should include clear color legends and larger, readable font sizes. Several elements, particularly those in white text, are difficult to interpret and should be improved for better visibility.
7. The KEGG and GO enrichment results are listed but not fully interpreted. Please explain how identified pathways relate mechanistically to PLOSL progression, especially microglial and osteoclast dysfunction.
8. The text states that PPARG is associated with disease progression but does not cite primary mechanistic literature. Please add supportive references.
9. The molecular docking results indicate moderate binding affinity, so the therapeutic relevance should be discussed cautiously. While the MD simulation plots (RMSD, RMSF, and Rg) are provided, the interpretation of these results should be expanded to clearly explain the structural stability and conformational behavior of the ligand–protein complexes.
10. The chemical structures of AMG-131 and Elafibranor should be included as figures to support clarity.
11. The authors should also acknowledge the discontinued clinical trial status of Elafibranor and the experimental development stage of AMG-131 to avoid implying that these compounds are clinically viable options at this stage.
12. The manuscript uses long sentences that reduce readability. Grammar, sentence structure, and transitions require careful language editing.
13. The Conclusion section should be moderated; currently it implies therapeutic development readiness. Please emphasize that experimental validation is still required.
14. The authors may consider briefly discussing alternative hub candidates identified (e.g., C1QA, AIF1) to balance interpretation and avoid appearing to pre-select PPARG.
15. The study would benefit from a short limitations paragraph summarizing dataset size, lack of clinical validation, and computational-only nature.
16. The Introduction provides general background on PLOSL but could be more concise. Several paragraphs repeat known disease characteristics. Reducing redundancy will improve focus.
17. The Introduction should state a clear research hypothesis and specific study objectives at the end. Currently, the transition into the aim of the study is broad and lacks direct specificity.
18. Authors are advised to incorporate more recent literature (within the past 5 years) on PPARG signaling in neuroinflammation and osteoclast regulation to better support the rationale for selecting PPARG as the hub gene in this study.
19. Authors are advised to justify the selection of the specific GEO datasets used in this study, particularly regarding sample relevance and availability. Additionally, the differential expression threshold should be clarified, as the stated FDR < 2 is not standard practice; please correct or explain the appropriate adjusted p-value cut-off for clarity and consistency.
20. The PPI network analysis describes the construction process, but network confidence score choices (0.4 or 0.75) vary in different parts of the text. Standardize and justify the confidence threshold.
21. The CytoHubba ranking selection uses degree only. Consider including at least one additional centrality parameter (e.g., closeness or betweenness) to strengthen hub gene selection.
22. The volcano plot and heatmap would benefit from labeling the key differentially expressed genes to enhance interpretability and allow readers to easily identify the most significant up- and down-regulated genes.
23. The GO/KEGG tables list terms effectively, but narrative interpretation is brief. Highlight the most biologically relevant pathways in the context of PLOSL pathogenesis.
24. The presentation of docking results compares binding scores, but no comparison is made with known strong PPARG ligands beyond Rosiglitazone. Including known ligand reference values would contextualize the strength of interactions.
25. The Results section occasionally shifts into speculative statements (e.g., therapeutic implications) that belong in Discussion. Please separate objective findings from interpretation.
